# Adapting Cord Blood Collection and Banking Standard Operating Procedures for HLA-Homozygous Induced Pluripotent Stem Cells Production and Banking for Clinical Application

**DOI:** 10.3390/jcm8040476

**Published:** 2019-04-08

**Authors:** Belén Alvarez-Palomo, Joaquim Vives, Ricardo P. Casaroli-Marano, Susana G. Gomez, Luciano Rodriguez Gómez, Michael J. Edel, Sergi Querol Giner

**Affiliations:** 1 Banc de Sang i Teixits, Edifici Dr. Frederic Duran i Jordà, Passeig Taulat 116, 08005 Barcelona, Spain; jvives@bst.cat (J.V.); rcasaroli@ub.edu (R.P.C.-M.); sugomez@bst.cat (S.G.G.); lrodriguez@bst.cat (L.R.G.); 2 Musculoskeletal Tissue Engineering Group, Vall d’Hebron Research Institute (VHIR), Universitat Autònoma de Barcelona, Passeig de la Vall d’Hebron 129-139, 08035 Barcelona, Spain; 3 Department of Medicine, Universitat Autònoma de Barcelona, Passeig de la Vall d’Hebron 129-139, 08035 Barcelona, Spain; 4 Department of Surgery, School of Medicine & Hospital Clinic de Barcelona, University of Barcelona, 08036 Barcelona, Spain; 5 Institute of Biomedical Research Sant Pau (IIB-Sant Pau), 08035 Barcelona, Spain; 6 Molecular Genetics and Control of Pluripotency Laboratory, Department of Biomedicine, Institute of Neuroscience, Faculty of Medicine, University of Barcelona, Casanova 143, 08036 Barcelona, Spain; edel.michael@gmail.com; 7 Victor Chang Cardiac Research Institute, Sydney, NSW 2145, Australia; 8 Harry Perkins Research Institute, Centre for Cell Therapy and Regenerative Medicine (CCTRM), School of Medicine and Pharmacology, University of Western Australia, Perth, WA 6009, Australia; 9 Department of Physiology, Anatomy and Genetics, Oxford University, Oxford OX3 7BN, UK; 10 Centro de Oftalmología Barraquer, Institut Universitari Barraquer, Universitat Autònoma de Barcelona, 08193 Barcelona, Spain

**Keywords:** SOP, cord blood bank, induced pluripotent stem cells, cell therapy, advanced therapies medicinal products, HLA haplobank

## Abstract

In this article, we will discuss the main aspects to be considered to define standard operation procedures (SOPs) for the creation of an induced pluripotent stem cell (iPSC) bank using cord blood (CB)—or similar cell type—bank guidelines for clinical aims. To do this, we adapt the pre-existing SOP for CB banking that can be complementary for iPSCs. Some aspects of iPSC manufacturing and the particular nature of these cells call for special attention, such as the potential multiple applications of the cells, proper explanation to the donor for consent of use, the genomic stability and the risk of genetic privacy disclosure. Some aspects of the iPSC SOP are solidly established by CB banking procedures, other procedures have good consensus in the scientific and medical community, while others still need to be further debated and settled. Given the international sharing vocation of iPSC banking, there is an urgent need by scientists, clinicians and regulators internationally to harmonize standards and allow future sample interchange between many iPSC bank initiatives that are springing up worldwide.

## 1. Introduction (From Cord Blood Banks into Induced Pluripotent Stem Cells Banks)

Umbilical cord blood (CB) has been used clinically since the late 1980s as a source of hematopoietic stem cells to treat blood disorders such as lymphomas and leukaemia [1]. Given this long history of clinical use, much can be learnt from CB banking for other more advanced cell therapies currently in development. CB banks (CBBs) were initially established for hematopoietic progenitors transplants [2,3], and large inventories of clinical-grade, HLA-typed CB units are stored all over the world. Having large inventories of clinical-grade CB cells stored for transplantation leads to considering the use of these units for alternative applications in cell therapy or in regenerative medicine that will also help the sustainability of public CBBs [4]. One of the proposed uses is the production of induced pluripotent stem cell (iPSC) lines from banked umbilical cord. In 2006, the seminal work of Yamanaka’s group described the creation of iPSCs that are cells with embryonic stem-cell-like features made from somatic adult cells [5]. Since then, the interest in stem cell research and therapies expanded exponentially, as the ethical concerns of embryonic stem cells (hESCs) were not applicable and the door to autologous treatment was opened [6]. Very soon after the discovery of iPSCs, it was argued that the time and financial costs of personalized iPSC production would be unaffordable in most cases and that the creation of an iPSC bank of homozygous HLA haplotypes could be an acceptable allogeneic approach [7]. The concept of a bank of HLA matching homozygous donors had already been proposed for embryonic stem cells before [8,9]. With just a few homozygous donors with wide HLA immunological compatibility among the population, a wide percentage of the population would be provided with the possibility of iPSC-derived therapies [10]. Consequently, the idea of repurposing CB banks for creating HLA-homozygous iPSC banks developed [11]. Cells from cord blood are good candidates as starting material for iPSCs because they are genetically young, CBB count on thousands of cryostored CB units are already HLA-typed, and protocols for reprogramming them into iPSCs are well established [12,13]. Rao and colleagues reviewed the success and opportunities arising from coupling CBBs to iPSC banks and how one could benefit the other [14]. Several CBBs have already set themselves to the task of creating an iPSC bank from the stored homozygous CB units. The Catholic University of South Korea described the generation of 13 clinical-grade HLA-homozygous iPSC lines made from several HLA-typed banked tissues, including cord blood [15] and more specifically, the Korean CHA Medical Centre described their experience repurposing the CB bank for haplobanking of HLA-homozygous iPSCs and the generation of 10 lines that would match 41.07% of the Korean population [16]. For other populations with higher diversity of HLA types, the number of haplotype cell lines required increases, though the benefits are still significant with a feasible number of iPSC cell lines. A study on the UK population estimated that 10 selected homozygous donors would provide a complete match on HLA-A, HLA-B and HLA-DR for 37.7% of the population, and a beneficial match (just one HLA-A or one HLA-B mismatch) for 67.4% [9], and for the highly ethnically diverse population of California, it was estimated that fewer than 80 haplolines would cover the needs of more than 50% of the population [17]. HLA-homozygous iPSC initiatives are starting in other countries such as Spain (IPS-PANIA project), led by the Banc de Sang y Teixits (BST) and CMRB, in Australia (Murdoch Children’s Research Institute) and in the UK (CGT Catapult initiative) that use peripheral blood. For that matter, the Global alliance for iPSC therapies (GAiT) was formed with the mission: “an international initiative to support the implementation and clinical application of therapies derived from induced pluripotent stem cells” and provides guidance and support for the creation and harmonization of iPSC banks globally.

**Figure 1:**
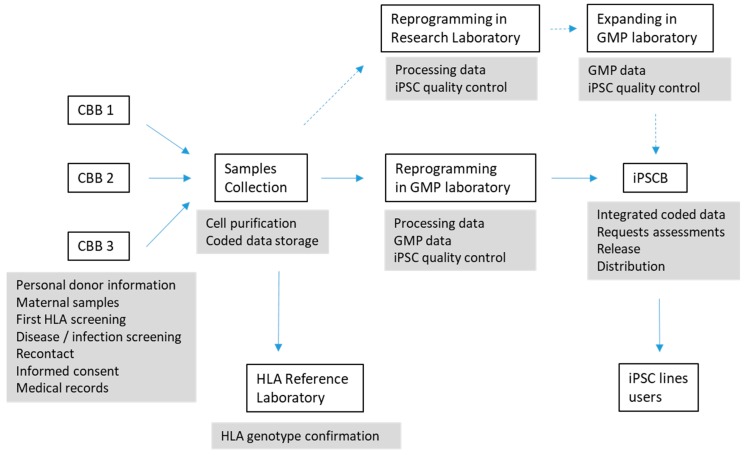
Different steps of the process of repurposing a cord blood bank (CBB) to iPSC bank (IPSCB) and identification of areas for the definition of SOP.

Creating a clinical-grade, Good Manufacturing Practice (GMP)-standard human iPSC bank with cell samples that are intended to be used by numerous users for both research and clinical applications is a complex process that has to be carefully thought about and well documented, with a quality management structure in place. This can cause work plans, applications, ethics and costs to differ greatly between iPSCs for research use or for clinical applications. When considering the ethics of iPSC applications, it overcame the dilemma of embryo destruction posed by hESC; however, other ethical issues still arise such as the risk of undesired differentiation of cells and malignant transformation, misuse for unauthorised reproductive cloning or animal–human chimera creation [18]. The International Stem Cell Banking Initiative (ISCBI) published an extensive article on the development of iPSC banks (or seed stocks) for clinical applications [19]. Specific policies, manuals, forms, registries and standard operating procedures (SOPs) have to be created. Figure 1 shows a schematic description of the process of creating an iPSC bank from CB banks’ samples and identifies the areas for which the different SOPs have to be created.

Many of the SOPs already existing for CBBs can be adapted to an iPSC bank (termed iPSCB). In order to help standardize policies and SOPs for CBBs, the Foundation for the Accreditation of Cellular Therapy and NETCORD published a document with guidelines for cord blood collection and banking describing the main considerations for the bank structure and operations and providing a list of relevant SOPs [20]. These documents can be useful for updating and revising CBB SOPs and offer a reference point in many aspects for creating a new SOP for iPSC banks (Table 1).

## 2. Operational Standards and Bank Organization

**Figure 2:**
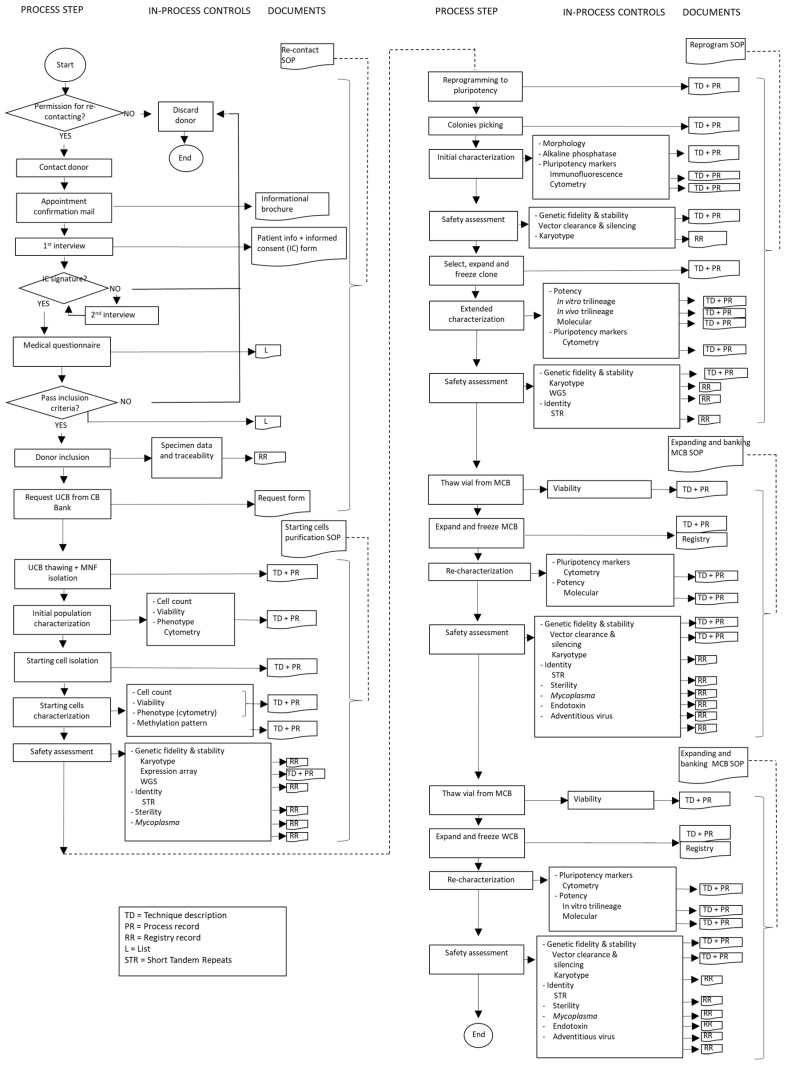
Flow chart of building an iPSCB from CBB and associated SOP and records.

From contacting donors to releasing a clinical-grade iPSC to an external partner for clinical use, a long list of steps have to be followed and each step involves a considerable number of documents to be created in the form of manuals, SOPs, records, lists and others that will assure the good management, functionality, traceability and accounting of the bank. Figure 2 shows an example of a work flow with associated documents for each step.

### 2.1. Bank Constitution and Juridical Status

#### 2.1.1. A New Cell Bank: iPSC Bank Is a New Legal and Administrative Entity

An HLA-homozygous iPSC bank will have a well-defined objective that is different from the CBB the original cells come from. Therefore, the iPSC bank should be created as a new legal and administrative entity. However, the specific formula for creating the new entity and the determination of the property of the cell lines will come down to each country and the national specific legal regulation for pluripotent cells. Private CBBs surpass public CBBs in number and total number of units, and their participation in constructing an iPSC cell bank would be of great interest; however, private CBBs have been created and marketed for private use rather than donation to the common good [12,21]. An HLA haplotype iPSC bank is conceived for an allogeneic use and the general opinion is that donors are not to be compensated or personally benefit from the donation [22]. Some of the existing policies and SOPs from the CBBs could be a guide for the iPSC bank; however, since the iPSC lines could have multiple applications such as research, clinical trials or starting material for a cell therapy, new SOPs will have to be created to deal with the lines requests, which will have to be considered one by one and will have to include the approval of an ethics committee. 

#### 2.1.2. Repurposing the Samples: To Be Carefully Registered and Documented

Using the identified homozygous HLA from the CBBs to create the iPSC lines means that the cells will not be used anymore for haematopoietic progenitor transplant as they were originally intended when donated. In any case, a unit from the cord blood bank would only be transferred to the iPSC bank if there is at least one other unit with the same haplotype, to ensure that future HSC recipients’ needs can be met. Some authors argue that making iPSCs from banked CB could be done without ultimately compromising their original use [14] since only a fraction of the cells are sufficient for efficient iPSC reprogramming. However, in practice, the numbers of hematopoietic progenitors are already not sufficient normally for one adult patient and taking away a fraction of the cells for reprogramming and another fraction to keep as quality control for the generated iPSCs will hardly leave enough cells in the sample for a successful transplant. Moreover, significant administrative and regulatory complications would be generated in the process. When using banked CB units (or other banked biospecimens) as starting material for making the iPSCs, the transfer of the CB units from the CB bank to the iPSC bank, besides being explicitly included in the donor informed consent, should be done with the permission of the legal health authorities that supervise the donors’ registry. The iPSC bank will create a new product status for the transferred CB unit.

In conclusion, the process of taking a sample from a CBB to be transformed and stored in an iPSC bank should be carefully registered and documented. New SOP and Registry documents should be put in place to sign a sample out of one bank and into another cell bank.

## 3. Donor Management and Samples Collection

### 3.1. Information Brochure for the Donor and Informed Consent: Significant Differences in the Nature of the Donation

The umbilical CB information for the donor and informed consent (IC) documents could be a basis for defining an iPSC bank IC form. There are significant differences in the nature of the cell donation compared to CB, mostly concerned with the long-term implications of iPSC lines such as: (i) wider possibilities for research and medicine, including multiple therapies and recipients, and (ii) still undefined purposes and commercial applications. Therefore, it is necessary to produce a new document in which the potential donor is informed of the characteristics of the new donation:

○ Uses for research and for transplantation into patients
○ Possibility of a large number of different applications (more than one indication)
○ Possibility of a large number of recipients
○ Commercial applications
○ Whole-genome sequencing (WGS) for quality control purposes
○ The possibilities of complete anonymization and of consent retrieval

Technically, the iPSC IC form is actually permission for transferring the already donated samples to be used for a different application from the original purpose. A CB donation IC form that includes the expressed acceptance of the donor to be recontacted paves the way for institutional permission to call back the donors and engage them in the new project. In this sense, it is advisable to include the possibility of recontact in the iPSC informed consent again, since new unforeseen possibilities might come up in the future that require recontacting the donor.

Normally, CB donors are given the possibility of withdrawing consent up to the point of transplantation. For iPSCs, on the contrary, many authors propose the possibility of withdrawal up to the point of creation of the iPSC line given the considerable investment in money and effort that is placed in creating one single line of clinical-grade iPSCs. In the same way, the possibility of delinking all donor medical information to the coded sample is more of a delicate issue when dealing with iPSCs compared to CB, since total anonymization would jeopardize many of the intended applications [23], especially for clinical trials and transplantation therapies, as it prevents retroactive validation. Complete anonymization does not completely guarantee anonymity and invalidates the sample for diagnostic purposes and, for quality reasons or for carrying on longitudinal and epidemiological studies, researchers might need to access the identity of the samples and link them to medical journals and register data [24].

#### 3.1.1. SOP for Recontacting and Obtaining the IC: Signing Procedures Are Valid and Legally Binding

The SOP should be defined for the sequence of events that takes place since recontacting the identified homozygous donor until signature of the IC. All the ethical and legal aspects should be considered so the donor feels well informed and not coerced, and so the IC form and signing procedure are valid and legally binding. Creating a detailed SOP is particularly important for the cases in which samples might be recruited from more than one CBB to create a single iPSC bank. The SOP for recontacting and IC signature should include the information brochure for the donor, the IC form, the documents for registering the follow-up of the process and a detailed description of the sequence of events and the persons responsible for each step. Once the potential homozygous donors are identified, they will be contacted and invited for a personal interview with a trained researcher or medical staff involved in the project to get a full explanation of the nature of the project and the implications of the sample cession. This interview should include an informative document and the donor should be given the possibility to ask the interviewer any question she might have about the project. Also, the donor should be offered the possibility of taking time to think about it and to consult with other people, who might even include the baby whose cord blood was stored, and whose genetic information is encoded in the cord blood cells. Therefore, the possibility of a second appointment should be offered to give enough time to the donor to consider the consent. Some donors could be hard to locate as they might have changed their contact details since the time of donation. However, the number of donors to be recontacted is relatively small, especially when covering the most frequent haplotypes, and possible back-up donors can be considered.

#### 3.1.2. Friendly Information for Donor Autonomy During IC: Best Care and Information Is Essential

The background information for the donor that accompanies the IC form should be carefully selected and laid out, not only due to the complexity of the scientific and technical aspects of the intended use of the donated cells, but also to cover the specific social sensibilities for each country. How a donor regards her identity and individuality, duty to the common good or altruistic willingness to donate cells can vary considerably in each society. Even the trust of the donor in the scientific and medical community can vary from place to place. Special emphasis should be placed on addressing all the key issues the donor should be aware of, using a register that is easily understandable for anyone who is not an expert in the field and without creating unnecessary alarm or suspicions. Making sure that the donor gets the best care and information is particularly important to respond to the donor spirit of altruism and generosity and to be in line with the values of transparency, excellence and service to society, as stated by *Banc de Sang i Teixits* and shared by all public blood and tissue banks.

#### 3.1.3. Harmonizing IC Internationally: Consensus Point that Should Be Present in All IC for Making iPSCs

Importantly, besides including all the local references to national legislation and cultural and religious particularities, it is of crucial importance that the IC for making iPSC banks be harmonized internationally. Some authors have published insightful considerations about consensus point that should be present in all ICs for making iPSCs [25], some even with the specific perspective of the case of using previously collected research biospecimens [25,26]. The European Bank for Induced Pluripotent Stem Cells (EBISC) has issued a donor information leaflet template and the global Alliance for iPSC Therapies (GAiT) will be delivering one soon too [27].

### 3.2. Health Questionnaires and Medical Records: Minimum Health Information Requirements Need to Be Established to Set the Sample Inclusion Criteria for iPSCs

CBB medical information on donors can be used for iPSC banks. An extensive health questionnaire is performed with CB donors during labour and shortly after delivery asking about mother and baby general health, hereditary diseases in the family, blood transmittable diseases and transmittable infections or risky behaviour. Some banks also use a postdelivery questionnaire at the time a recipient for haematopoietic stem cell transplantation is identified. In this case, new information about the mother’s health should be acquired at the point of donor selection. In cases where the questionnaire was done a long time before the use of cells for reprogramming, updated information on the mother’s and, particularly, on the child’s health should be collected, in particular information referring to the appearance of serious diseases or genetic origin conditions. Similar SOPs, forms and data storage and protection that were used for the CBB can be used for the iPSC bank. In some cases in which the child might be of age at the time of recontacting, he or she might have some reticence on his/her mother providing information on his/her health. In this case, minimum health information requirements should be established to set the sample inclusion criteria to be part of the iPSC bank. In order to preserve maximum confidentiality, personal information that can directly identify the donor should be kept in the institution in which the original donation was done. The iPSC bank should be transferred the codified medical records and other relevant information with the maximum protocols of data confidentiality.

### 3.3. Specimen Collection and Donor Eligibility: Step-by-Step Consideration

Collection of CB at delivery and the subsequent specimen acceptance are well documented processes in a CBB, with specific policies, SOPs and instructions that have to take into account the collection process and the mother and infant health. This process involves a large number of health professionals such as nurses, obstetricians and laboratory qualified personnel. In the case of an iPSC bank, the specimen collection step would be the one requesting specific HLA-homozygous samples from the CBB collection, simplifying the number of SOPs, registries and training material involved in this step.

Regarding donor eligibility, several articles with step-by-step consideration for making clinical-grade iPSCs describe the criteria for CB eligibility emphasizing the importance of reviewing the medical records, physical exams and clinical laboratory testing for relevant communicable disease agents or diseases [19,28]. In the cases where the samples came from a CBB, these considerations were already taken into account when the CB units were accepted in the CBB. The test for viral blood-borne pathogens (HIV, HBV, HCV, CMV and others emerging or endemic to the donor’s origin) on maternal samples as donor surrogate are mandatory in Europe according to the Cell and Tissue Directive (Directive 2004/23/CE). If data and techniques used are up-to-date, this information could be used for the iPSC bank.

### 3.4. Genotyping for HLA: Harmonize the Information from CB to iPSC

When umbilical CB units are included in the CBB registries, HLA typing is required. CB banks have been run for long periods of time and the degree of resolution can vary and often low-resolution techniques like serological, and more recently, DNA-based methods such as sequence-specific oligonucleotide probes (SSOP) or sequence-specific primer (SSP) PCR can coincide. When the specimen was identified as a suitable donor, a higher resolution method was used such as sequence-based typing (SBT) using Sanger sequencing. However, the introduction of Next-Generation Sequencing (NGS) in the determination of HLA haplotypes has united high throughput with high resolution. New developments allow for HLA determination at very high resolution of big numbers of samples simultaneously, for a low price [29,30]. Therefore, given the advantages and universalisation of NGS for HLA typing, this is probably the method of choice for HLA confirmation of the homozygous before being selected for reprogramming and inclusion in the iPSC bank. Therefore, SOPs describing the HLA retyping of the HLA-homozygous CB units should be put in place to harmonize the information in all the samples.

For the HLA-homozygous selection, a projection of coverage of the target population needs to be done, and to do this, access to a large database of HLA samples is necessary. It is important to keep a registry of all the associated documents from ethics committees, IC, data privacy and so forth, associated with the use of the database, and compliance should be reflected in the SOPs for iPSC use.

### 3.5. Notification of Relevant Clinical Data and Counselling: New Information Regarding Potential Health Threats with iPSC

CBBs have in place SOPs to notify positive or indeterminate results of (i) infectious diseases, (ii) genetic testing and (iii) the event of unexpected adverse effects in a transplant recipient. The quality controls performed with iPSC-generated data may reveal new information regarding potential health threats for the donor, so specific policies and SOPs should be created for this purpose. Since the samples will be coded in the iPSC bank and only the original CBB that provided the original cells have the donor personal information, the iPSC bank must actually notify the CBB and there, the medical coordinating staff should decide about donor notification and counselling.

## 4. iPSC Production

### 4.1. Cell Reprogramming to iPSC: With GMP-Compliant Processes in Clinical-Grade Facilities

Unlike CB banking that involves nonsubstantially manipulated stored specimens, the manufacturing of iPSC lines is a more complex process requiring not only well-defined cell lines but also a very extensive cellular characterization, viral and microbial status determination and product potency assay to show identity, safety and pluripotency capacity after manipulation, respectively.

Though there are no international established standards for clinical-grade iPSCs at the time of writing this review, iPSC lines—or more precisely, iPSC-derived cell products—fall under the definition of Advanced Therapy Medicinal Products [31,32] and as such, these should be produced following Good Manufacturing Practices (GMPs). Several publications have set up the discussion about these standards [19,23,33] and previous literature on hESCs can be of guidance for the iPSC field [34,35].

From our point of view, there would be at least two possible approaches to create a clinical-grade iPSC bank in accordance with current regulations in EU:

1. Performing the whole process, reprogramming of iPSC lines, downstream manipulations and cryopreservation, under cGMP conditions.
2. “Cleansing” already reprogrammed iPSCs into cGMPs.

The first approach represents what is the classic pharmaceutical implementation of manufacturing processes of medicinal products but involves the inversion of a high quantity of resources that few institutions consider affording at least until some therapeutic therapy based on these cells have clearly shown to work.

The second one represents a strategy similar to some hESC lines that have been previously used in a clinical setting and for which starting material was isolated in research-grade conditions and later transitioned into GMPs [36].

In this regard, existing European guidelines on GMPs specific to ATMPs on article 7.35 [37] refer to this issue and seem to consider that, in exceptional cases and after evaluation and explicit approval of competent authorities, the design of a battery of quality control tests and specifications able to assure the quality of this kind of starting material could give rise to a regulatory acceptance to be further used to manufacture an ATMP.

In view of the above, the “cleanse” of iPSCs manipulated out of GMP compliance approach should be suitable for well-characterized cell lines made during research and development investigations, implying complex and highly specialized manipulations not within the reach of much of the research teams and thus representing a material nearly impossible to replace with other ones that are GMP-compliant.

In the case that iPSCs are partially produced in a non-GMP-compliant environment, the use of xeno-free and feeder-free processes and an exhaustive traceability of all the steps and raw material will be fundamental for later regulatory approval as a medicine as well as following specific requirements for raw materials of biological origin for the production of cell-based therapy medicinal products. It is essential to create a “Cell Line History” file for each iPSC in the bank, to keep a history of the cells. The clinical utility of an iPSC line could be compromised if some information or documents are not present in the file. The International Stem Cell Banking Initiative (ISCBI) published a list of typical information that might be required in a cell line history [30]. In any case, the final expansion and stock production, quality control and storage should be made with GMP-compliant processes in clinical-grade facilities.

All the steps of the reprogramming and expansion processes should be carefully documented and traceability assured. SOPs, procedure descriptions, registries and listings should be written for every step of the process, batch records, facilities and instrumentation maintenance and supply chain guarantees. It is also necessary to define specifications (quality and security requirements) that should be met by any ATMP manufacturer eventually interested in using the iPSC as starting material. Also, back-up suppliers for every reagent and material should be provided. If a part of the process takes place in a non-GMP facility, still an effort should be placed in keeping records and documentation collection as extensive as possible and the risks and implications of the specifications of the future ATMP should be evaluated and registered.

### 4.2. Cell Characterization Pipeline

Though acquisition of self-renewal and pluripotency capacity assays are quite well generalized in the scientific community, there is still no international guideline that will serve as reference for iPSC banks and for regulators to accept them as starting material for therapies, although considerable effort is being put into it internationally by the scientific community, health institutions and commercial companies [38]. The International Stem Cell Banking Initiative and GAIT have issued consensus guidelines that are useful reference for characterization and quality control and safety [19], including reference to applicable pharmacopeial methods when available [33]. There is consensus in the assessment of Alkaline Phosphatase activity and of the expression of classical pluripotency markers by flow cytometry, such as OCT3/4, NANOG, SSEA4, TRA-1-60, TRA-1-81 [33,37], though protein expression by immunofluorescence has widely been used in the field. For differentiation capacity, formation of tissue of the three germ layers—endoderm, mesoderm and ectoderm—is an obvious must, but whether in vivo teratoma formation is required or in vitro embryoid bodies formation or directed differentiation is sufficient still remains to be settled. Pluripotency markers and three germ layers gene expression at the molecular level are still not viewed as mandatory, although the now-available standardized commercial kits for this purpose (hPSC Scorecard^TM^ or Pluritest^TM^) would make harmonization and evaluation much easier. SOPs for genomic instability have not been considered and are discussed in more detail below.

### 4.3. Quality Control and Safety: Long-Term Clinical Success of iPSCs Needs Stringent Guidelines of Genetic Stability

Some mandatory safety tests are common to the products in a CBB, such as microbiological sterility (virus, bacteria and mycoplasma), endotoxin or pyrogenicity, and there are established tests institutionally recognized. In the case that the iPSCs have been cultivated with antibiotics, additional validations should be put in place for the sterility tests (test suitability).

Unlike CB cells, which are minimally manipulated before storing, the process of reprogramming into iPSCs involves overexpression of the reprogramming factors, several weeks in cell culture and the reprogramming process, which itself causes great stress to the cells.

Regarding the reprogramming method, different reprogramming methods have been used to create iPSCs such as retroviral vectors, lentiviral vectors, plasmids, mRNA or recombinant proteins [39]; however, nonintegrative methods—to avoid the risk of insertional mutagenesis—are the choice when it comes to making clinical-grade iPSCs, in particular, mRNA, Sendai virus or episomal vectors [40]. In any case, the use of exogenous recombinant nucleic acids makes these three approaches fall into gene therapy category for the regulatory agencies [41].

Altogether it is mandatory to include a series of safety tests that include karyotyping for gross chromosomal abnormalities, Short Tandem Repeats (STRs) for identity and RT-PCR for residual vector clearance. Vector clearance, especially of viral origin vectors, is an important aspect to settle to later go through regulatory approval and at the moment there are no accredited laboratories offering this assay. Therefore, special care should be taken by each lab in developing this assay, taking into account a standard curve, an internal reference and two different regions [33].

Genomic stability is a particularly concerning issue to the iPSC field. If the field of human iPSC is to have long-term clinical success in transplantation in humans, then stringent guidelines to the level of genetic stability analysis including WGS need to be considered. Many reports have raised the concerns over the genomic stability of iPSCs, especially when the cells are intended for clinical use, and in many cases for life-long engraftment into the patient [42]. The first clinical trial using iPSC-derived cells, the RIKEN trial in Japan [43], stopped the assay in the second patient since three single-nucleotide variations (SNVs) and three copy-number variants (CNVs) were found in the iPSC line that were not present in the patient’s original cells before reprogramming [44]. Therefore, single-nucleotide polymorphisms (SNPs) and comparative genomic hybridization (CGH) arrays have been proposed as regular tests before releasing iPSC lines into clinical use to detect changes in the genome at higher resolutions, like CNV. Moreover, NGS has allowed WGS at a more affordable price and the detection of genomic alterations at the nucleotide level. However, the threshold of genomic alterations for the acceptance of the iPSC in the clinical-grade bank is still to be settled. Although a huge amount of information can be deduced with arrays and NGS, the general acceptance is that high-resolution genetic analysis is necessary before clinical approval of iPSC lines [45]. Further consideration needs to be given to the interpretation of the fitness of an iPSC line and its tumorigenic potential extracted from WGS or CNV data obtained with these assays and where to set the acceptance threshold. Consequently, it is a matter of debate if high-resolution assays are to be included in the quality and safety control SOP for an iPSC bank.

## 5. Data Management and Sample Release

### 5.1. Data Collection and Confidentiality: How and When to Retrace to the Original Donor Information for iPSC Bank

When the specimens to be reprogrammed are directly obtained for creating an iPSC bank, the SOP should describe the personal and medical data collection and registration in a way similar to how they are collected for a CBB, including donor contact data, medical records and a full description of material procurement. When the cells come from a previous bank, as would be the case for a CBB to become part of the iPSC bank, the personal information identifying the particular donor should stay with the original bank and only the coded medical and basic information (date of birth, country of origin) stored in the iPSC bank. However, careful planning on how and when to retrace to the original donor should be reflected in the SOP with data protection.

Specific SOPs will have to be generated to account for the confidentiality of the genetic data that is generated with STR fingerprinting and WGS and that could eventually lead to identification of the original donor and be potentially misused. The new European General Data Protection Regulations, effective since 2018, have implications to anyone who generates banks or distributes human-derived iPSCs and associated data and it is strengthening the focus on accountability, transparency and data minimization [46]. Hopefully, best practice standards for genetic data management for iPSC banks will be soon standardized and available [47].

### 5.2. Sample Release: Only Released as Stated in the Informed Consent

Besides the technical SOP describing the freezing, storage and transportation processes for the bank specimens, additional aspects have to be considered in the sample release SOP. Unlike CBBs, which aim to be a source of hematopoietic progenitors for a limited number of blood disorders, with a few newer applications (plasma or endothelial progenitors), the intended uses of the iPSCB samples by researchers and clinicians are numerous and many yet unknown. It is important to set up all the procedures and documentation that will assure that the samples are only released as stated in the informed consent originally signed by the donors, which might include some limitations on the use of the cells or a revision of the projects by an Ethics Committee.

## 6. International Harmonization of HLA-Homozygous iPSC Procurement and Production: Facilitates Clinical Use of iPSC-Derived Cell Therapy Products

HLA-homozygous iPSC banks would be particularly useful if international cooperation is in place, since the effort to create enough lines to cover all the national population is mostly unattainable by a single blood cell bank for most countries and sample sharing can substantially increase the coverage for a country. Also, for clinical trials and commercial applications, harmonizing and standardizing the quality and characteristics of the iPSC lines in the different banks are particularly relevant. Therefore, special emphasis is focused on standardization of iPSC production, characterization and quality control of the iPSC produced worldwide. Several initiatives, for example, Global Alliance for iPSC Therapies, are working in this direction. Table 2 shows the most relevant legislation concerning the production of an iPSC bank for clinical use. International harmonization of national or regional legislations and guidelines is greatly expected to facilitate the clinical use of iPSC-derived cell therapy products.

## 7. Summary: Impact on Future Developments

Repurposing donated cord blood units and developing high-quality and diversified iPSC lines are feasible. Adaption of CB SOPs to create international SOPs for iPSC clinical use has been proposed in this review. Qualified CB banks and CB units are essential for starting material for developing tissue-specific stem cell therapies in an allogeneic setting using HLA matching. The large availability of validated CB units (it is estimated that more than 700,000 public CB units are available worldwide) makes it possible to identify the appropriate haplotypes that cover a defined population. Aggregation of all ethnic groups will ensure equal access to the iPSC therapy for all patients in need.

The stringent quality assurance programs in CB banking will help the development of high-quality and diverse iPSC cell lines. Use of standardized protocols will allow the desired strategy to generate a worldwide iPSC banking registry. The addition of cell lines with particular haplotypes will help academic and commercial developers in the goal of generating tissue-specific stem cells that will contribute to the translation of regenerative medicine to treat human disease.

## Figures and Tables

**Table 1:** Possible adaptation of required Policies and SOPs from the NetCord-FACT International Standards for CB collection, banking and release for administration [20].

Subject		Standard
CBB	iPSC Bank	
Donor recruitment		B3.1.1, C3.3.1
Informed consent		B3.1.3, C3.1.3
Donor eligibility criteria and determination		B3.1.5, C3.4.6
Interaction between the CB collection site and the CBB	Interaction between CBBs and the iPSC bank	B3.1.6
Infant donor health		B3.1.7, C3.1.6, C3.1.7
Storage of CB units, associated samples, maternal samples and documentation at the CB collection site	Storage of iPSC aliquots, associated samples and documentation associated to original CB units	B3.1.10, C6.7
Transport and shipping of the CB unit, associated samples, maternal samples and documentation to the CB processing facility	Transport and shipping of the CB unit, associated samples and documentation to the iPSC production centre	B3.1.41, C3.1.13
Labelling of the CB unit, associated samples, reference samples, retention samples, maternal samples and associated documents at the CB collection site and CB processing facility	Labelling of the iPSC aliquots, associated samples, reference samples, retention samples and associated documents at the original CBB and iPSC production centre	B3.1.14, C3.1.10, C6.5, D2.1.4
CB unit acceptance criteria for receipt, processing, cryopreservation and storage		B3.1.15, B3.1.19, D2.1.1, D2.1.7,
Process control, including product specifications and nonconforming products		B3.1.16, B3.1.21, C3.1.11, D2.1.3, D2.1.8
Storage of reference samples, retention samples and maternal samples for testing	Storage of reference samples and retention samples for testing	B3.1.17, D4.1.12, D4.1.2
Communicable disease testing, microbial cultures, hemoglobinopathy testing and other testing.		B3.1.18, B3.1.19, D2.1.7
Notification of mothers or their responsible physicians and/or governmental agencies, when required, of positive or indeterminate communicable disease and/or genetic test results		B3.1.20
Listing, search, selection, reservation, release and distribution of CB units	Listing, search, selection, reservation, release and distribution of iPSC aliquots	B3.1.23
HLA typing		B3.1.25, D2.1.9, E3.2.1
Data management		B3.1.30
CB unit records	iPSC lines records	B3.1.31, C3.1.17, D2.1.11
CB unit disposition	iPSC lines disposition	B3.1.32, C3.1.18, D2.1.12

**Table 2:** Current legislation concerning the production of cell-based therapies.

	EU	USA	Japan
Tissue procurement	EU Tissues and Cells Directive 2004/23/EC. EU Blood Directive 2002/98/EC	FDA 21 CFR 1271 Human Cells, Tissues and Cellular and Tissue-Based Products	The Act on Safety of Regenerative Medicine. MHLW (25/11/2014)
GMP	Directive 2003/94/EC GMP for medicinal and investigational products for human use	FDA 21 CFR 201 Subchapters D (Drugs for Human Use) and F (Biologicals)	
Clinical use	EU Tissues and Cells Directive 2004/23/EC	FDA 21 CFR 50, 56, 210, 312, 314, 320, 812, 814	
Quality and safety	EU Tissues and Cells Directive 2004/23/EC	FDA 21 CFR 600, 601, 610	Guidelines on Ensuring the Quality and Safety of Products Derived from Processed HUMAN Stem Cells MHLW No. 0907-2,3,4,5,6

## Abbreviations

ATMP	Advance therapy medicinal product
BST	Banc de Sang i Teixits (Blood and Tissue Bank of Catalonia)
CB	Cord blood
CBB	Cord blood bank
CGH	Comparative genomic hybridization
CNV	Copy number variation
EBISC	European bank for induced pluripotent stem cells
GAiT	Global alliance for iPSC therapies
hESC	Human embryonic stem cells
HLA	Human leukocyte antigen
IC	Informed consent
iPSC	Induced pluripotent stem cells
IPS PANIA	Spanish consortium to create a clinical-grade haplobank of iPSC lines
ISCBI	International stem cell banking initiative
NGS	Next-generation sequencing
RT-PCR	Real-time polymerase chain reaction
SNV	Single-nucleotide variation
SOP	Standard operation procedure
WGS	Whole-genome sequencing
